# Evaluation of PD-L1 and TIM-3 Pathways in T Cells During Experimental Bovine Leukemia Virus Infection in Sheep

**DOI:** 10.3390/vetsci12090810

**Published:** 2025-08-26

**Authors:** Wisa Tiyamanee, Tomohiro Okagawa, Shinji Yamada, Mari Ikehata, Hayato Nakamura, Maho Inoue, Naoya Maekawa, Yukinari Kato, Shiro Murata, Kazuhiko Ohashi, Kenji Murakami, Satoru Konnai

**Affiliations:** 1 Department of Disease Control, Faculty of Veterinary Medicine, Hokkaido University, Sapporo 060-0818, Japan; 2 Business Development Unit, FASMAC Co., Ltd., Atsugi 243-0021, Japan; 3 Cooperative Department of Veterinary Medicine, Faculty of Veterinary Medicine, Iwate University, Morioka 020-8550, Japan; 4 Department of Antibody Drug Development, Tohoku University Graduate School of Medicine, Sendai 980-8575, Japan; 5 Veterinary Research Unit, International Institute of Zoonosis Control, Hokkaido University, Sapporo 001-0020, Japan; 6 International Affairs Office, Faculty of Veterinary Medicine, Hokkaido University, Sapporo 060-0818, Japan; 7 Institute for Vaccine Research and Development (HU-IVReD), Hokkaido University, Sapporo 001-0021, Japan; 8 One Health Research Center, Hokkaido University, Sapporo 060-0818, Japan

**Keywords:** sheep, PD-L1, TIM-3, bovine leukemia virus, immune suppression

## Abstract

Bovine leukemia virus (BLV) infects B cells in ruminants and causes lymphoma after an extended latency period. Previous studies have demonstrated T-cell exhaustion through the upregulation of immunoinhibitory molecules, including programmed death-ligand 1 (PD-L1) and T-cell immunoglobulin and mucin domain-3 (TIM-3), in BLV-infected cattle. However, studying T-cell exhaustion across all BLV infection stages remains challenging due to the virus’s prolonged latency in cattle. Sheep provide a valuable model, as they develop lymphoma more rapidly than cattle. This study examined PD-L1 and TIM-3 expression kinetics and T-cell function in BLV-infected sheep. During persistent infection, PD-L1 expression was correlated with BLV proviral load. TIM-3 expression increased in CD4^+^, CD8^+^, and γδTCR^+^ T cells. Functional analysis revealed that TIM-3 blockade enhanced T-cell activation markers (CD25 and CD69) in cultured PBMCs from infected sheep and increased CD69^+^IFN-γ^+^ and CD69^+^TNF-α^+^ populations, particularly among CD4^+^ T cells. Combined PD-L1 and TIM-3 blockade significantly enhanced cytokine production in both CD4^+^ and CD8^+^ T cells, while PD-L1 blockade alone showed limited effects. These findings demonstrate the effect of TIM-3 blockade in restoring immune function during chronic BLV infection, effective both alone and in combination. This study validates sheep as a valuable model for investigating immune checkpoint dynamics and evaluating immunotherapies for BLV infection and other chronic diseases.

## Simple Summary

Immunoinhibitory molecules, such as programmed death-ligand 1 (PD-L1) and T-cell immunoglobulin and mucin domain-3 (TIM-3), contribute to T-cell exhaustion in chronic infections. In a sheep model of bovine leukemia virus (BLV) infection, PD-L1 expression was correlated with BLV proviral load, and TIM-3 expression was upregulated in multiple T-cell subsets. Moreover, TIM-3 blockade, alone or combined with PD-L1 inhibition, enhanced T-cell activation and cytokine production, while PD-L1 blockade alone had limited effects. These findings demonstrate that TIM-3 plays a role in T-cell dysfunction in sheep and support the use of sheep as a model for evaluating immunotherapies targeting immunoinhibitory molecules in BLV infection and other chronic infectious diseases.

## 1. Introduction

Exposure to chronic infections and malignant tumors frequently leads to T-cell exhaustion, characterized by the development of functionally impaired T cells [1,2,3,4]. This phenomenon is mediated by immunoinhibitory molecules, including programmed cell death-1 (PD-1) [5,6], programmed death-ligand 1 (PD-L1) [7,8], and T-cell immunoglobulin and mucin domain-3 (TIM-3) [9,10]. PD-L1, a co-inhibitory transmembrane protein, plays a critical role in suppressing immune responses and promoting self-tolerance by modulating T-cell activity and inducing the apoptosis of antigen-specific T cells [6,11]. When PD-L1 binds to PD-1, it suppresses the proliferation of PD-1-positive T cells, inhibits their cytokine secretion, and induces apoptosis [6,11]. PD-L1 also plays a crucial role in immune evasion by dampening antitumor immune responses [6,11]. TIM-3 is a type I transmembrane protein expressed on various immune cells, including CD4^+^ and CD8^+^ T cells [12]. Similar to the PD-1/PD-L1 pathway, TIM-3 is associated with T-cell exhaustion. However, TIM-3 expression is more extensive, spanning a broad range of immune cells beyond T cells [10,13,14,15,16]. Furthermore, TIM-3 binds to multiple ligands, including galectin-9 [17], phosphatidylserine, high-mobility group box 1 (HMGB-1) [18], and carcinoembryonic antigen-related cell adhesion molecule 1 (CEACAM-1) [19]. Therefore, this pathway operates through a distinct immune suppression mechanism compared to the PD-1/PD-L1 pathway.

While immunotherapies targeting immunoinhibitory molecules have demonstrated promising efficacy and durable clinical benefits in cancer treatment [11,20,21], approximately half of patients do not respond to anti-PD-1 monotherapy [22,23]. Additionally, a previous study reported that TIM-3 expression increases in T cells following anti-PD-1 monotherapy [24], suggesting that resistance to PD-1 blockade may involve compensatory upregulation of alternative inhibitory pathways, such as TIM-3. Consequently, combination blockade therapies targeting multiple immunoinhibitory molecules are being investigated to achieve better therapeutic outcomes than monotherapy [25].

In veterinary medicine, bovine leukemia virus (BLV) infection is a major cause of immunosuppression in cattle [26]. BLV is a retrovirus that infects B cells in ruminants and causes enzootic bovine leukosis (EBL), a fatal B-cell lymphoma, in some infected cattle [27]. Most infected cattle remain asymptomatic and are classified as aleukemic (AL). However, approximately 30% of infected cattle develop persistent lymphocytosis (PL), characterized by benign proliferation of circulating B cells. Because BLV integrates into the host’s chromosomal DNA, this cellular expansion facilitates viral propagation [27]. Fewer than 5% of infected cattle progress to EBL, typically after prolonged latency periods of 5–10 years [27]. BLV-induced immunosuppression has a significant economic impact by increasing the risk of lymphoma development, decreasing milk production, heightening susceptibility to secondary infections, and shortening animal lifespans [28,29,30,31]. BLV prevalence varies globally, reaching as high as 100% in some regions [32].

Our previous studies have demonstrated that BLV infection leads to the upregulation of immunoinhibitory molecules PD-1, PD-L1, and TIM-3, and that blocking these molecules can restore T-cell function both in vitro and in vivo [33,34,35]. However, studying BLV immunopathogenesis in cattle remains challenging due to the extended latency period and the small proportion of infected cattle that develop EBL. As a result, the precise immunomodulatory dynamics during BLV infection remain incompletely understood.

Sheep provide an effective experimental model for studying various chronic bovine infections, including BLV infection [36,37,38,39]. Compared to cattle, sheep develop these diseases more rapidly and at higher rates [36,39], which results in larger experimental groups and improved cost-effectiveness of immunological studies. However, the limited availability of sheep-specific immunological reagents has hindered progress in this field. We recently addressed this limitation by confirming that anti-bovine PD-L1 antibodies cross-react with ovine orthologs [40]. Therefore, this study aims to investigate T-cell exhaustion mediated by immunoinhibitory pathways in BLV infection using sheep as an experimental model.

## 2. Materials and Methods

### 2.1. Blood Sample Collection and Experimental BLV Infection in Sheep

Heparinized peripheral blood samples were collected from ten BLV-uninfected healthy sheep (Crossbreed) housed at a private farm in Japan. Informed consent for the use of blood samples for this study was obtained from an animal owner.

Experimental BLV infection in sheep was conducted at the Research Farm of the Field Science Center, Faculty of Agriculture, Iwate University. Seven sheep (Friesland or Crossbreed, one to four months old) were intraperitoneally inoculated with 3.0 × 10^7^ BLV-infected leukocytes isolated from BLV-infected cattle. Four infected animals were used in all analyses, including temporal analyses, and the other three infected animals were enrolled in the experiment at a later stage. Four age-matched animals (crossbreed) were maintained as BLV-negative controls. Heparinized peripheral blood samples were collected at least once every six months before and after viral challenge.

All animal experiments were approved by the Iwate University Animal Care and Use Committee (approval number A202112, approval date 19 February 2021). All procedures in this study were in accordance with the ARRIVE guidelines [41].

### 2.2. Cell Isolation

Peripheral blood mononuclear cells (PBMCs) were isolated from heparinized blood samples using Percoll density gradient centrifugation (GE Healthcare, Chicago, IL, USA). Cells were washed three times with phosphate-buffered saline (PBS; pH 7.2) containing 0.5 mg/mL disodium ethylenediaminetetraacetic acid (EDTA) (Dojindo Molecular Technologies, Kumamoto, Japan) and were then resuspended in PBS. PBMCs were used immediately for analysis or stored at −80 °C until needed.

### 2.3. Nucleic Acid Extraction and Proviral Load Measurement

Total DNA was extracted from isolated PBMCs using the Quick-DNA Miniprep Kit (Zymo Research, Irvine, CA, USA) according to the manufacturer’s instructions. DNA concentration and purity were measured using a NanoDrop 8000 spectrophotometer (Thermo Fisher Scientific, Waltham, MA, USA). BLV proviral loads were quantified using a BLV Detection Kit targeting the BLV *tax* gene (Takara Bio, Shiga, Japan) and real-time PCR on a LightCycler 480 System (Roche Diagnostics, Mannheim, Germany) according to the manufacturer’s instructions.

### 2.4. Flow Cytometric Analysis of PD-L1 and TIM-3 Expression

PD-L1 expression on IgM^+^ B cells and TIM-3 expression on CD4^+^, CD8^+^, and γδTCR^+^ T cells were analyzed by flow cytometry using the antibodies described below. Frozen PBMCs were thawed and incubated in blocking buffer consisting of PBS supplemented with 10% inactivated goat serum (Thermo Fisher Scientific), 0.5% bovine serum albumin (Sigma-Aldrich, St. Louis, MO, USA), and 2 mM disodium EDTA (Dojindo Molecular Technologies) at 25 °C for 15 min.

For PD-L1 staining, cells were incubated with anti-bovine PD-L1 monoclonal antibody (mAb) (6C11-3A11; rat IgG2a [42]) or rat IgG2a isotype control (R35-95; BD Biosciences, San Jose, CA, USA) at 25 °C for 20 min. Cells were then washed and stained with allophycocyanin (APC)-conjugated anti-rat immunoglobulin antibody (Southern Biotech, Birmingham, AL, USA), Alexa Fluor 488-labeled anti-bovine IgM mAb (PIG45A; mouse IgG2b; VMRD, Pullman, WA, USA), and Fixable Viability Dye eFluor 780 (Thermo Fisher Scientific) at 4 °C for 20 min. PIG45A mAb was pre-labeled with Alexa Fluor 488 using the Zenon mIgG2b Labeling Kit (Thermo Fisher Scientific). Stained cells were washed and analyzed using a BD FACSLyric flow cytometer (BD Biosciences).

For TIM-3 staining, cells were incubated with anti-bovine TIM-3 mAb (1D3; mouse IgG1 [43]) or mouse IgG1 isotype control (15H6; Southern Biotech) at 25 °C for 20 min. Cells were then washed and stained with Alexa Fluor 647-conjugated anti-mouse IgG antibody (Thermo Fisher Scientific), Alexa Fluor 488-labeled anti-ovine CD4 mAb (17D; mouse IgG1; Washington State University Monoclonal Antibody Center, Pullman, WA, USA), phycoerythrin (PE)-conjugated anti-bovine CD8 mAb (CC63; mouse IgG1; Bio-Rad, Hercules, CA, USA), PE/cyanine7 (PE/Cy7)-conjugated anti-ovine γδTCR mAb (86D; mouse IgG1; Washington State University Monoclonal Antibody Center), and Fixable Viability Dye eFluor 780 (Thermo Fisher Scientific) at 4 °C for 20 min. The 17D mAb was pre-labeled with Alexa Fluor 488 using the Zenon mIgG1 Labeling Kit (Thermo Fisher Scientific). The 86D mAb was conjugated with PE/Cy7 using the Lightning-Link Conjugation Kit (Abcam, Cambridge, UK). Stained cells were washed and analyzed by flow cytometry as described above.

### 2.5. T-Cell Marker and Cytokine Production Analysis

PBMCs (1 × 10^6^) were resuspended in 250 μL of RPMI 1640 medium (Sigma-Aldrich) supplemented with 10% heat-inactivated fetal bovine serum (Thermo Fisher Scientific), 100 IU/mL penicillin, 100 μg/mL streptomycin, and 2 mM L-glutamine (Thermo Fisher Scientific). Cells were stimulated with concanavalin A (Con A, 0.1 μg/mL) in the presence of anti-bovine PD-L1 mAb (4G12; 10 μg/mL [44]), anti-bovine TIM-3 mAb (1D3; 10 μg/mL [43]), rat IgG2a isotype control (2A3; 10 μg/mL; Bio X Cell, Lebanon, NH, USA), or mouse IgG1 isotype control (15H6; 10 μg/mL; Southern Biotech) at 37 °C with 5% CO_2_ for 18 h. For cytokine detection, brefeldin A (2.5 μg/mL; Sigma-Aldrich) was added to the culture 6 h before harvest. After incubation, culture supernatants were collected and stored at −20°C until analysis.

For T-cell marker analysis, cultured PBMCs were collected and incubated in blocking buffer as described above. Cells were then washed and stained with Alexa Fluor 488-conjugated anti-ovine CD4 mAb (17D; Washington State University Monoclonal Antibody Center), peridinin-chlorophyll-protein/cyanine5.5 (PerCP/Cy5.5)-conjugated anti-bovine CD8 mAb (CC63; Bio-Rad), PE-conjugated anti-bovine CD25 mAb (CACT116A; VMRD), Alexa Fluor 647-conjugated anti-bovine CD69 mAb (KTSN7A; Washington State University Monoclonal Antibody Center), and Fixable Viability Dye eFluor 780 (Thermo Fisher Scientific) at 4 °C for 20 min. The 17D, CACT116A, and KTSN7A mAbs were pre-labeled with Zenon Mouse IgG1 Labeling Kits (Thermo Fisher Scientific). The CC63 mAb was conjugated with PerCP/Cy5.5 using the Lightning-Link Conjugation Kit (Abcam).

For intracellular cytokine detection, cultured PBMCs were collected and incubated in blocking buffer as described above. Cells were then stained with Alexa Fluor 488-conjugated anti-ovine CD4 mAb (17D; Washington State University Monoclonal Antibody Center), PerCP/Cy5.5-conjugated anti-bovine CD8 mAb (CC63; Bio-Rad), and Fixable Viability Dye eFluor 780 (Thermo Fisher Scientific) at 4 °C for 20 min. After surface staining, cells were fixed and permeabilized using Fixation Buffer and Intracellular Staining Perm Wash Buffer (BioLegend, San Diego, CA, USA) according to the manufacturer’s protocol. Cells were then stained with PE-conjugated anti-bovine interferon gamma (IFN-γ) mAb (CC302; Bio-Rad) and biotin-conjugated anti-bovine tumor necrosis factor alpha (TNF-α) mAb (CC328; Bio-Rad) at 4 °C for 20 min. Cells were then washed and incubated with PE/Cy7-conjugated streptavidin (BioLegend) at 4 °C for 20 min. The 17D and CC63 mAbs were conjugated as described above. Stained cells were analyzed by flow cytometry as described above.

### 2.6. Statistical Analysis

Statistical analyses were performed using GraphPad Prism version 10.1.1 for macOS (GraphPad Software, Boston, MA, USA). The Mann–Whitney U test was used for PD-L1 and TIM-3 expression analysis. The Friedman test followed by Dunnett’s multiple comparisons test was used for T-cell marker and cytokine expression analysis by flow cytometry. Statistical significance was defined as *p* < 0.05.

## 3. Results

### 3.1. Cross-Reactivity of Anti-Bovine PD-L1 and TIM-3 MAbs with Ovine PD-L1 and TIM-3

**Figure 1:**
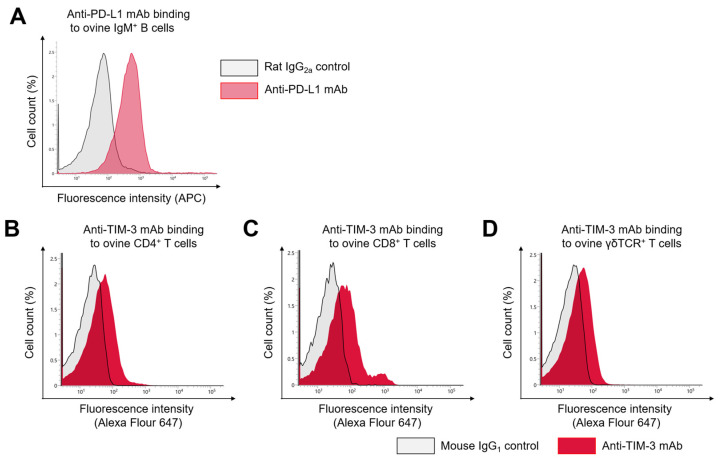
Cross-reactivity of anti-bovine PD-L1 and TIM-3 monoclonal antibodies in ovine PBMCs. (**A**) Cross-reactivity of anti-bovine PD-L1 monoclonal antibody (mAb) with fresh ovine PBMCs. The gray histogram shows fluorescence intensity of the rat IgG2a isotype control. The pink histogram shows staining with anti-bovine PD-L1 mAb. All data were gated on live IgM^+^ cells within PBMCs. (**B**–**D**) Cross-reactivity of anti-bovine TIM-3 mAb with fresh ovine PBMCs. The gray histograms show fluorescence intensity of the mouse IgG1 isotype control. The red histograms show staining with anti-bovine TIM-3 mAb. Histograms were gated on CD4^+^ (**B**), CD8^+^ (**C**), and γδTCR^+^ T cells (**D**). Gating strategies for the flow cytometric assays were shown in Supplementary Figure S1.

To validate our detection strategy, we tested anti-bovine PD-L1 (6C11-3A11; [42]) and anti-bovine TIM-3 mAbs (1D3; [43]) for cross-reactivity with ovine PBMCs. Both mAbs successfully recognized PD-L1 on IgM^+^ B cells and TIM-3 on CD4^+^, CD8^+^, and γδTCR^+^ T cells in ovine PBMCs without prior stimulation, confirming their suitability for subsequent expression analysis by flow cytometry (Figure 1A–D).

### 3.2. TIM-3 Blockade Enhances T-Cell Activation in Healthy Sheep

**Figure 2:**
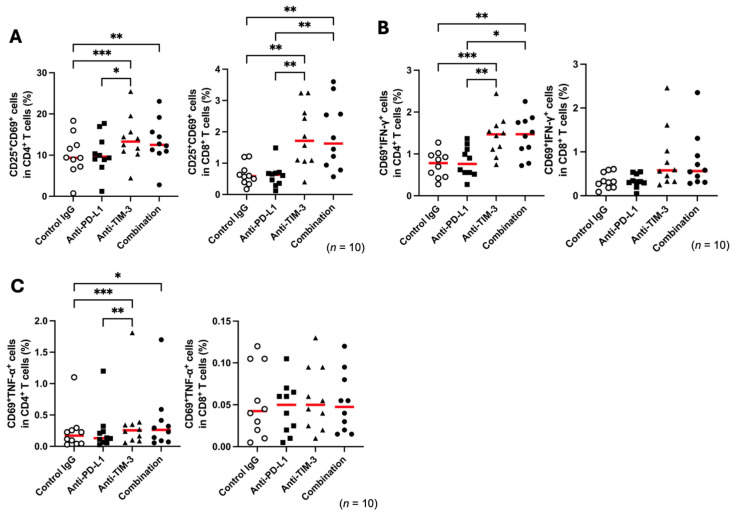
Blockade effects of anti-bovine PD-L1 and TIM-3 monoclonal antibodies in PBMCs of healthy sheep. (**A**–**C**) T-cell functional analysis in PBMCs from BLV-uninfected healthy sheep (*n* = 10) with blockade by anti-PD-L1 and anti-TIM-3 mAbs under ConA stimulation. (**A**) Frequency of activated T cells (CD25^+^CD69^+^) within CD4^+^ and CD8^+^ T-cell subsets. (**B**,**C**) Frequency of cytokine-producing activated cells (CD69^+^IFN-γ^+^ and CD69^+^TNF-α^+^) within CD4^+^ and CD8^+^ T-cell subsets. Gating strategies for the flow cytometric assays are shown in Supplementary Figures S2 and S3. Each symbol represents data from an individual animal. Red lines indicate median values. Significant differences compared between treatment groups: * *p* < 0.05, ** *p* < 0.01, *** *p* < 0.001.

Single and combined blockade of PD-L1 and TIM-3 pathways has been shown to enhance antiviral T-cell immunity in cattle [35]. Here, we examined whether PD-L1 and TIM-3 blockade similarly activate ovine T cells in cell culture assays using PBMCs from healthy sheep. To evaluate T-cell activation in sheep, we confirmed that ConA stimulation upregulated the expression of lymphocyte activation markers CD25 and CD69 on CD4^+^ and CD8^+^ T cells from healthy sheep (Supplementary Figure S4A,B). TIM-3 blockade further increased CD25^+^CD69^+^ cell populations in both CD4^+^ and CD8^+^ T cells under ConA stimulation, while PD-L1 blockade showed no similar effect (Figure 2A). Additionally, the proportions of CD69^+^IFN-γ^+^ and CD69^+^TNF-α^+^ cells increased in CD4^+^ T cells following TIM-3 blockade, but no significant increase was observed in CD8^+^ T cells (Figure 2B,C). Therefore, in T cells from healthy sheep, only TIM-3 inhibition had immune-activating effects.

### 3.3. Upregulation of PD-L1 Expression and Proviral Load Following BLV Infection in Sheep

**Figure 3:**
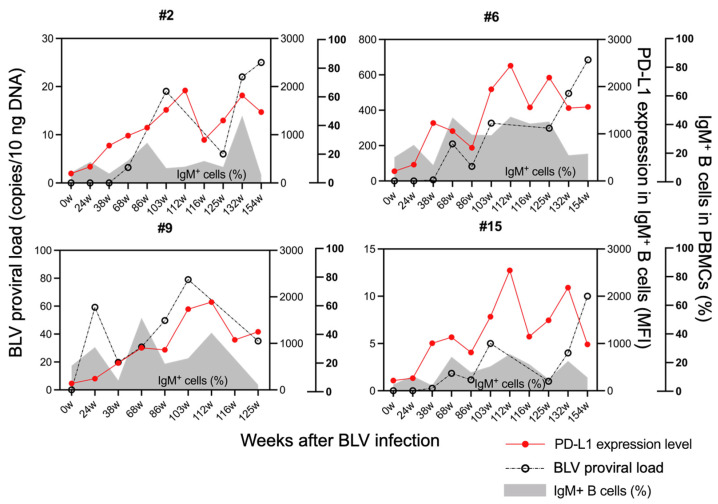
Kinetics of PD-L1 expression and proviral load in experimentally BLV-infected sheep. Temporal changes in BLV proviral load and median fluorescence intensity (MFI) of PD-L1 expression are shown for experimentally BLV-infected sheep (*n* = 4) at specified time points post-inoculation (weeks). Sheep #9 was excluded from this experiment at 132 weeks after BLV infection due to the low viability of isolated PBMCs. Gating strategies for the flow cytometric assays are shown in Supplementary Figure S1. Red lines and dots indicate PD-L1 MFI values. Black lines and dots indicate proviral load values. The gray shaded area indicates the baseline proportion of IgM^+^ B cells in PBMCs.

In BLV-infected cattle, PD-L1 expression reportedly increases in BLV-infected B cells during late disease stages (PL or EBL), contributing to T-cell exhaustion [33,34]. To investigate temporal changes in BLV-infected cells and immunoinhibitory molecules during the progression of BLV infection, we examined proviral loads and PD-L1 expression in B cells from the peripheral blood of experimentally infected sheep over time. Six months post-infection, PD-L1 expression levels in IgM^+^ B cells remained low in all animals but subsequently increased over time in parallel with rising proviral loads (Figure 3). These animals had not yet developed lymphoma, indicating that PD-L1 upregulation occurs gradually from early stages of BLV infection.

### 3.4. Upregulation of PD-L1 and TIM-3 in BLV-Infected Sheep

**Figure 4:**
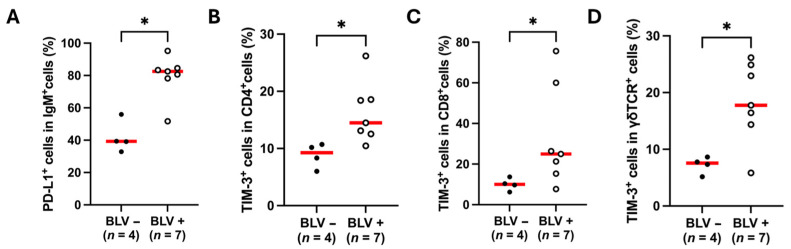
Comparison of immunoinhibitory receptor expression between healthy and experimentally BLV-infected sheep. (**A**) Percentage of PD-L1^+^ cells among IgM^+^ B cells in BLV-uninfected (*n* = 4) and BLV-infected sheep (*n* = 7). (**B**–**D**) Comparison of TIM-3^+^ cell frequencies in CD4^+^, CD8^+^, and γδTCR^+^ T-cell subsets between BLV-uninfected (*n* = 4) and BLV-infected sheep (*n* = 7). Gating strategies for the flow cytometric assays are shown in Supplementary Figure S1. Each dot represents data from an individual animal. Red lines indicate group median values. * *p* < 0.05.

Next, we compared the proportion of PD-L1-positive B cells between BLV-infected and BLV-uninfected sheep. The proportion of PD-L1^+^IgM^+^ B cells was significantly higher in BLV-infected sheep compared to uninfected sheep (Figure 4A). Our previous studies have shown that expression of immunoinhibitory molecules such as TIM-3 increases in T cells of BLV-infected cattle compared to uninfected cattle, correlating with T-cell exhaustion [35,43]. Consistent with findings in cattle, the proportion of TIM-3^+^ cells was higher in CD4^+^ and CD8^+^ T cells from BLV-infected sheep compared to uninfected animals (Figure 4B,C). Furthermore, previous studies have suggested that TIM-3 upregulation may destabilize γδ T cells and induce their dysfunction during chronic disease progression [16,45,46]. Accordingly, γδTCR^+^ T cells in BLV-infected sheep also exhibited elevated TIM-3 expression (Figure 4D).

### 3.5. Blockade of TIM-3 Pathway Restores T-Cell Function in BLV-Infected Sheep

**Figure 5:**
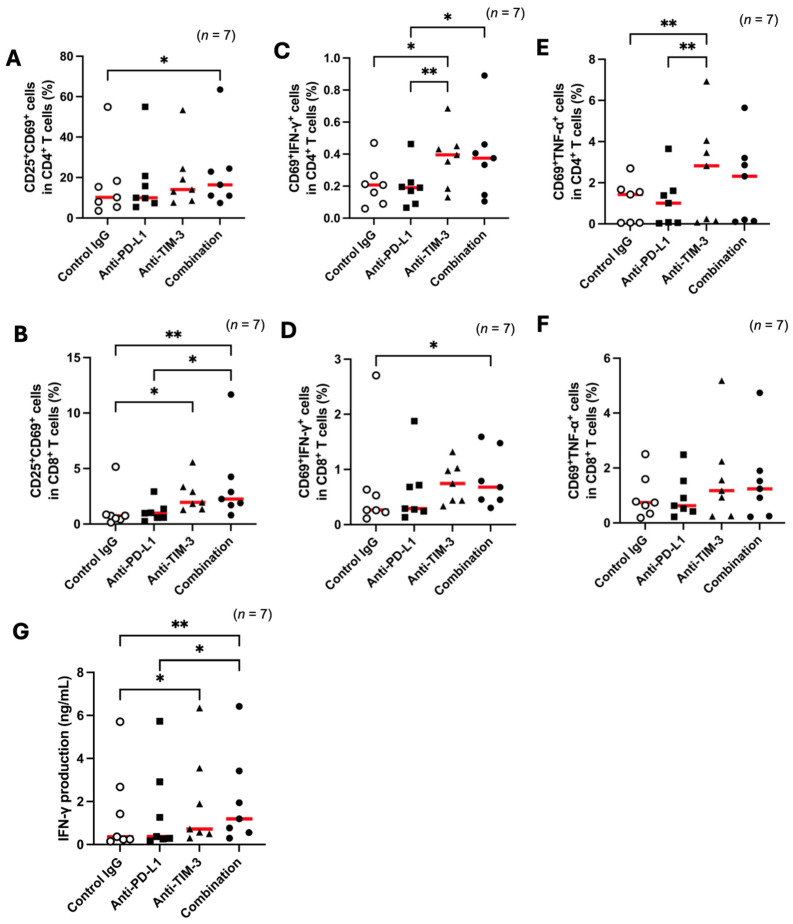
T-cell activation and cytokine responses in BLV-infected sheep following immune checkpoint blockade. (**A**,**B**) Frequencies of activated CD25^+^CD69^+^ cells in CD4^+^ and CD8^+^ T-cell subsets in PBMCs from BLV-infected sheep (*n* = 7) following ConA stimulation with blockade by anti-PD-L1 and anti-TIM-3 mAbs. (**C**–**F**) Frequencies of cytokine-producing CD69^+^ cells expressing IFN-γ (**C**,**D**) or TNF-α (**E**,**F**) under the same blockade conditions. (**G**) IFN-γ concentration of PBMCs from BLV-infected sheep (*n* = 7) following ConA stimulation with blockade by anti-PD-L1 and anti-TIM-3 mAbs. Gating strategies for the flow cytometric assays are shown in Supplementary Figures S2 and S3. Each dot represents data from an individual animal. Red lines indicate median values. Significant differences compared between treatment groups: * *p* < 0.05, ** *p* < 0.01.

In healthy sheep, TIM-3 blockade enhanced T-cell activation, while PD-L1 blockade showed no effect (Figure 2A–C). Given the increased expression of PD-L1 and TIM-3 observed in BLV-infected sheep, we performed cell culture assays using PBMCs from infected animals to investigate the immune-activating effects of TIM-3 and PD-L1 blockade. TIM-3 inhibition alone increased the CD25^+^CD69^+^ cell population in CD8^+^ T cells but not in CD4^+^ T cells (Figure 5A,B). However, dual blockade of PD-L1 and TIM-3 significantly increased the CD25^+^CD69^+^ cell population in CD4^+^ T cells (Figure 5A). Regarding cytokine production, both TIM-3 blockade and dual blockade significantly increased the proportions of CD69^+^IFN-γ^+^ and CD69^+^TNF-α^+^ cells in CD4^+^ T cells (Figure 5C,E). In CD8^+^ T cells, CD69^+^IFN-γ^+^ cells increased significantly with TIM-3 and dual blockade, whereas CD69^+^TNF-α^+^ cells did not (Figure 5D,F). These results indicate that TIM-3 blockade promotes T-cell activation in BLV-infected sheep and that combining it with PD-L1 blockade further enhances T-cell function.

## 4. Discussion

Over the past decade, immunotherapy has attracted increasing attention for its potential to treat both cancer and chronic infectious diseases. Immune checkpoint inhibitors (ICIs) have become a cornerstone of cancer immunotherapy, complementing conventional treatments such as surgery, chemotherapy, and radiotherapy. ICIs have demonstrated high response rates, particularly when used in combination regimes [47,48]. In veterinary medicine, PD-L1 blockade has also demonstrated favorable outcomes in some canine cancer patients, particularly those with oral malignant melanoma [49]. However, ICI monotherapy appears insufficient for most canine cancer patients. Koyama et al. reported that treatment with anti-PD-1 antibodies induces compensatory upregulation of other immune checkpoints, particularly TIM-3 [24]. This suggests that adaptive resistance to PD-1 blockade may develop through activation of alternative immunoinhibitory pathways.

In parallel with cancer immunotherapy, we have also explored the use of ICIs to treat chronic infectious diseases in livestock [40,42]. Previous studies in cattle have shown that ICIs targeting PD-L1 and TIM-3 can effectively restore T-cell function during BLV infection [35]. Therefore, we investigated the expression kinetics of PD-L1 and TIM-3 using the sheep model of BLV infection.

Previous studies in cattle reported increased PD-L1 expression on IgM^+^ B cells during late-stage BLV infection [33]. Our findings in BLV-infected sheep support and extend this observation, demonstrating that PD-L1 upregulation in IgM^+^ B cells correlates with increasing proviral load over time. To our knowledge, this study represents the first concurrent analysis of PD-L1 and TIM-3 expression in sheep during chronic viral infection. TIM-3 has been recognized as a key immunoinhibitory molecule contributing to T-cell exhaustion in various chronic infections [10,50,51,52,53].

TIM-3 inhibition enhanced expression of activation markers (CD25 and CD69) and cytokine production (IFN-γ and TNF-α) in both CD4^+^ and CD8^+^ T cells, while PD-L1 blockade alone showed minimal effects. We previously confirmed that the anti-PD-L1 mAb (4G12) used in the blockade assays in this study effectively inhibits ovine PD-1 and PD-L1 binding [40]. These findings suggest that TIM-3 may play a more dominant role than the PD-1/PD-L1 pathway in suppressing T-cell responses during chronic BLV infection, potentially reflecting a shift toward alternative exhaustion pathways as infection persists.

Furthermore, the marked upregulation of TIM-3 in γδTCR^+^ cells suggests that this subset also undergoes immune suppression. Given the role of γδ T cells in antiviral defense, their TIM-3-mediated dysfunction may contribute to viral persistence and immune evasion. Therefore, targeting TIM-3 could restore not only conventional T-cell responses but also the innate-like immunity mediated by γδ T cells [16,45,46].

In cattle, several studies have confirmed that PD-1 and TIM-3 synergistically induce T cell exhaustion during BLV infection, and the combination inhibition could enhance the antiviral effects against BLV in vitro [35,43]. In this study, PD-L1 inhibition showed minimal efficacy; however, whether this relates to PD-1 expression status in BLV-infected sheep remains unclear. We previously tested the cross-reactivity of our established anti-bovine PD-1 mAbs [34], but their reactivity with sheep PBMCs was insufficient to detect PD-1 expression by flow cytometry. A previous study in BLV-infected cattle showed that the expression level of PD-1 on T cells is associated with IFN-γ production by the blockade of PD-1/PD-L1 pathway [34]. Similarly, low levels of PD-1 expression could influence the inhibitory effects of PD-L1 in the sheep model. Therefore, the dominance of the TIM-3 pathway in T-cell exhaustion in sheep should be concluded after investigating PD-1 expression. Further research is needed to elucidate the immunosuppression mechanisms mediated by the PD-1/PD-L1 pathway in both sheep and cattle [54].

Despite this limitation, using sheep as an experimental model offers significant advantages. Compared to cattle, sheep exhibit faster disease progression and enable higher experimental throughput, reducing both time and cost when studying chronic infectious diseases. Our findings provide valuable translational data that bridge the gap between the natural bovine host and the experimental sheep model. These insights may guide future development of ICI-based immunotherapies for BLV infection and potentially other chronic diseases across different species.

## 5. Conclusions

During experimental BLV infection in sheep, PD-L1 and TIM-3 expression levels are upregulated in B cells and T cells, respectively. Functional assays revealed that TIM-3 blockade, alone or with PD-L1 blockade, enhanced T-cell activation and cytokine production, while PD-L1 inhibition had minimal effect. These findings underscore the central role of TIM-3 in T cell exhaustion and support the use of sheep as an experimental model for studying immunoinhibitory molecules throughout the infection stages and for assessing immunotherapies for BLV infection and other chronic diseases.

## Data Availability

The original contributions presented in this study are included in the article/Supplementary Material. Further inquiries can be directed to the corresponding authors.

## Supplementary Materials

The following supporting information can be downloaded at https://www.mdpi.com/article/10.3390/vetsci12090810/s1, Figure S1: Gating strategies of flow cytometric assays for the expression analysis of PD-L1 and TIM-3. Figure S2: Gating strategies of flow cytometric assays for the activation markers on T cells. Figure S3: Gating strategies of flow cytometric assays for the activation marker and cytokine production on T cells. Figure S4: Evaluation of T-cell activation marker under ConA stimulation in the cultivation of PBMCs from healthy sheep.

## Abbreviations

The following abbreviations are used in this manuscript:

AL	Aleukemic
APC	Allophycocyanin
BLV	Bovine leukemia virus
ConA	Concanavalin A
Cy5.5	Cyanin5.5
Cy7	Cyanin7
EBL	Enzootic bovine leukosis
EDTA	Ethylenediaminetetraacetic acid
ICI	Immune checkpoint inhibitors
IFN-γ	Interferon gamma
mAb	Monoclonal antibody
MFI	Median fluorescence intensity
PBMC	Peripheral blood mononuclear cells
PBS	Phosphate-buffered saline
PD-1	Programmed death protein 1
PD-L1	Programmed death-ligand 1
PE	Phycoerythrin
PerCP	Peridinin-chlorophyll-protein
PL	Persistent lymphocytosis
TIM-3	T-cell immunoglobulin and mucin domain-3
TNF-α	Tumor necrosis factor alpha
