# Supplementary material for: Evaluation of PD-L1 and TIM-3 Pathways in T Cells During Experimental Bovine Leukemia Virus Infection in Sheep
Source: Vet Sci. 2025 Aug 26;12(9):810. doi: 10.3390/vetsci12090810 (PMC12474299; doi:10.3390/vetsci12090810)
Supplement: Supplementary file 1 [file vetsci-12-00810-s001.zip › vetsci-3758298-supplementary.pdf]

Supplementary Figure 1

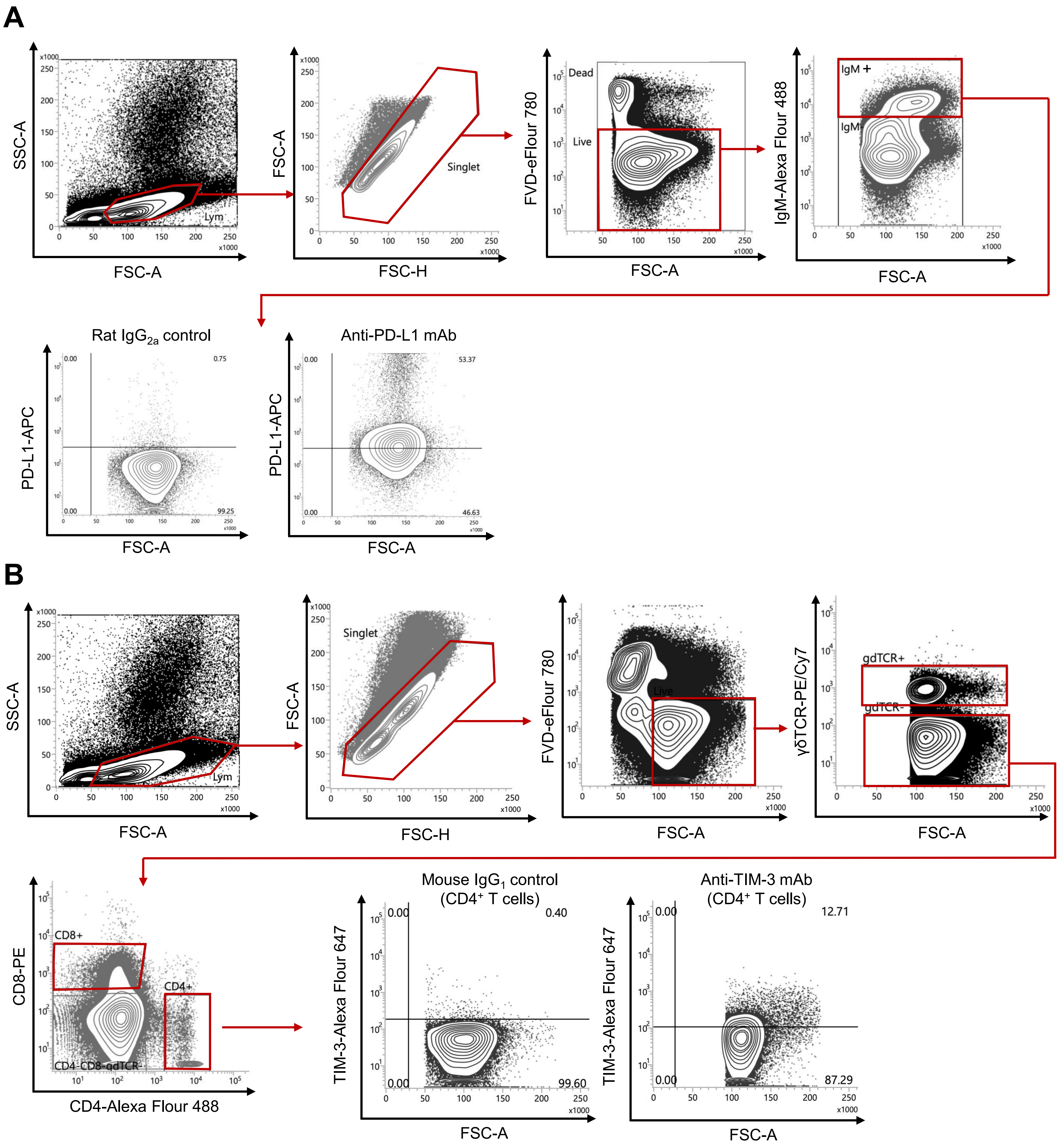

Figure S1. Gating strategies of flow cytometric assays for the expression analysis of PD-L1 and TIM-3.

(a, b) Representative plots of the flow cytometric assays for PD-L1 expression on IgM^{+} B cells (a) and TIM-3 expression on CD4^{+}, CD8^{+}, and γδTCR^{+} T cells. Red squares and polygons identify the gated population. Red arrows show the flow of gating strategies to use the gated population for the next gating.

Supplementary Figure 2

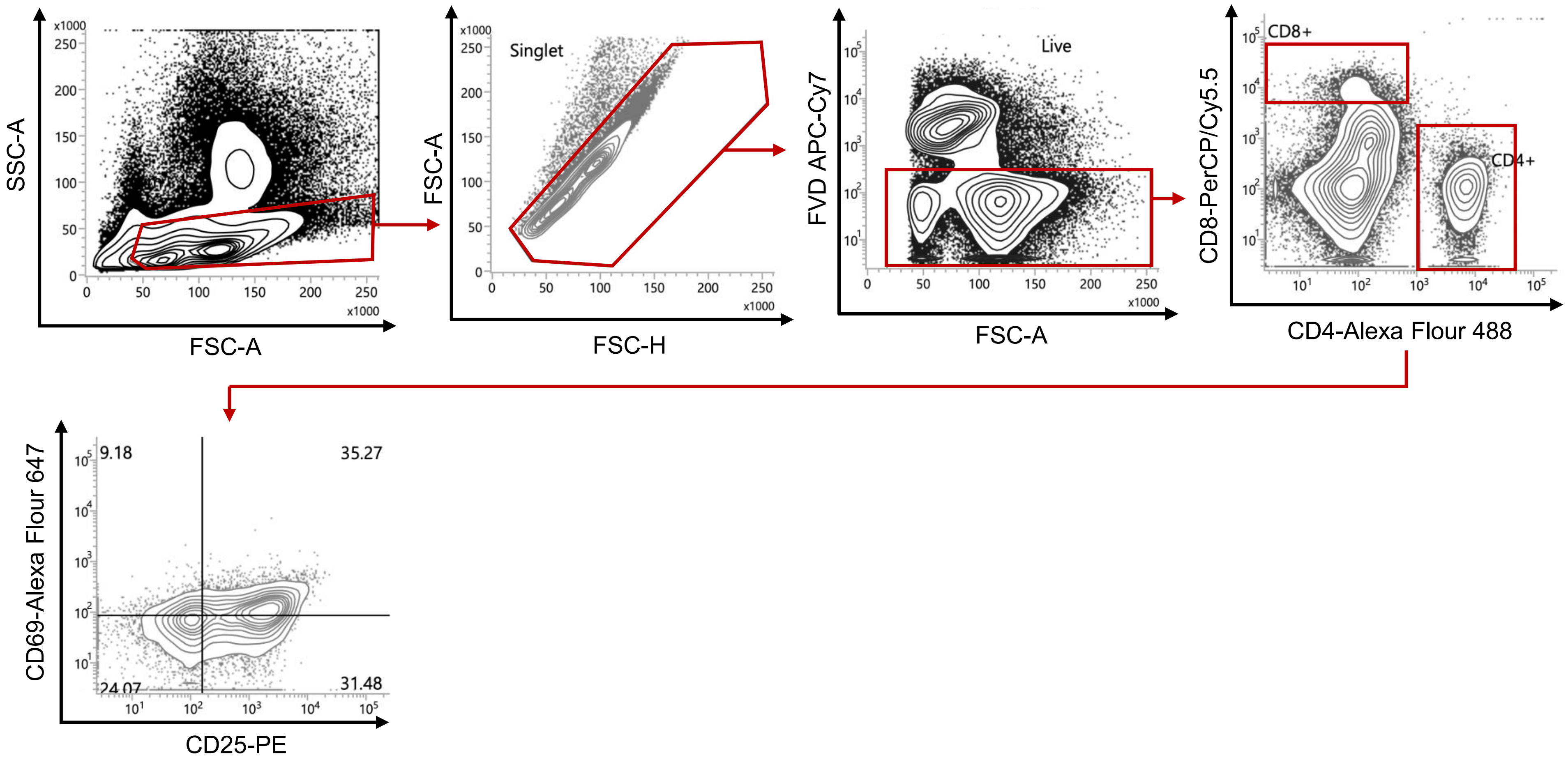

Figure S2. Gating strategies of flow cytometric assays for the activation markers on T cells.

Representative plots of the flow cytometric assays for CD25 and CD69 expression on $CD4^{+}$ and $CD8^{+}$ T cells. Red squares and polygons identify the gated population. Red arrows show the flow of gating strategies to use the gated population for the next gating.

Supplementary Figure 3

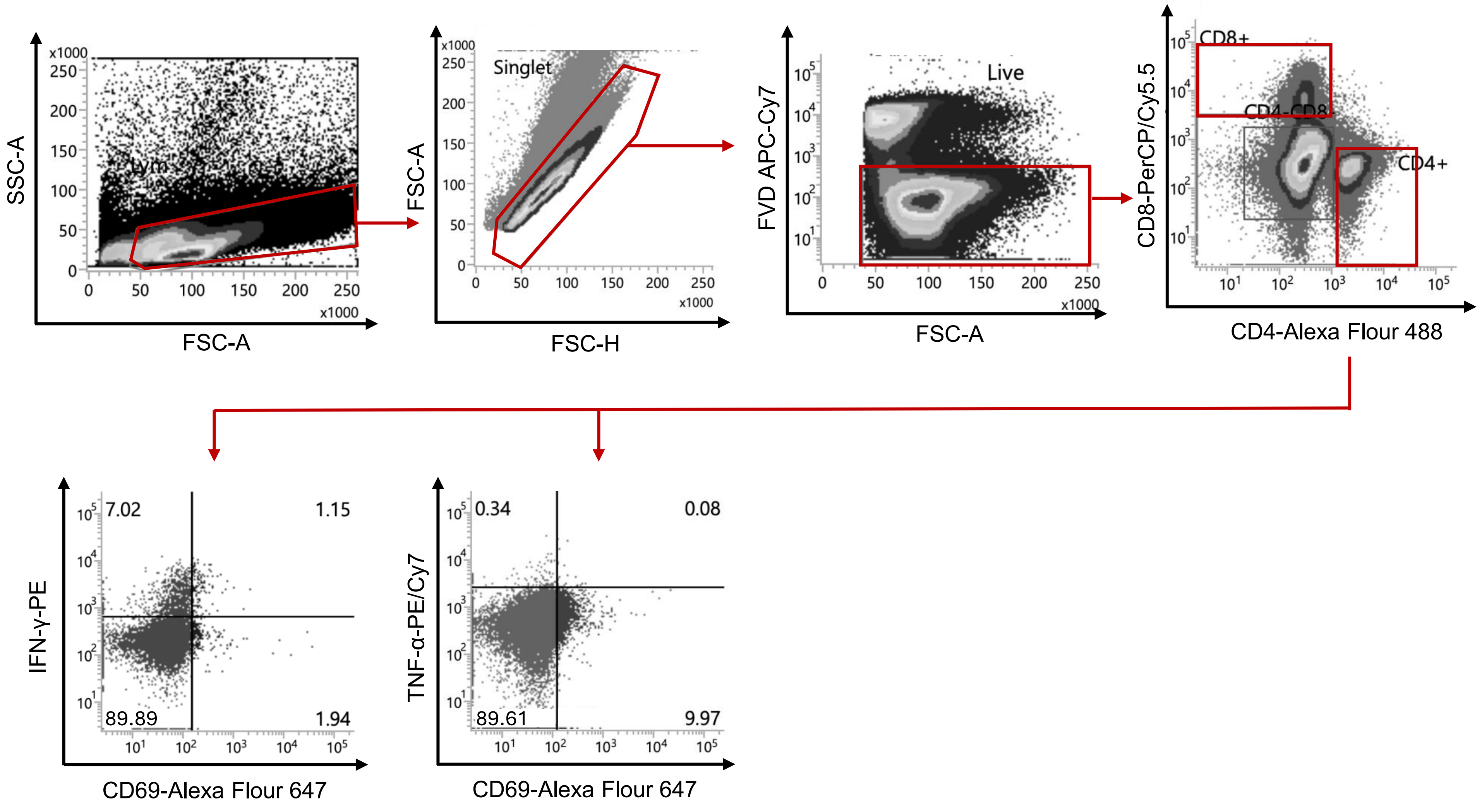

Figure S3. Gating strategies of flow cytometric assays for the activation marker and cytokine production on T cells.
Representative plots of the flow cytometric assays for CD69 expression and cytokine production (IFN-γ and TNF-α) on $CD4^+$ and $CD8^+$ T cells. Red arrows show the flow of gating strategies to use the gated population for the next gating.

Supplementary Figure 4

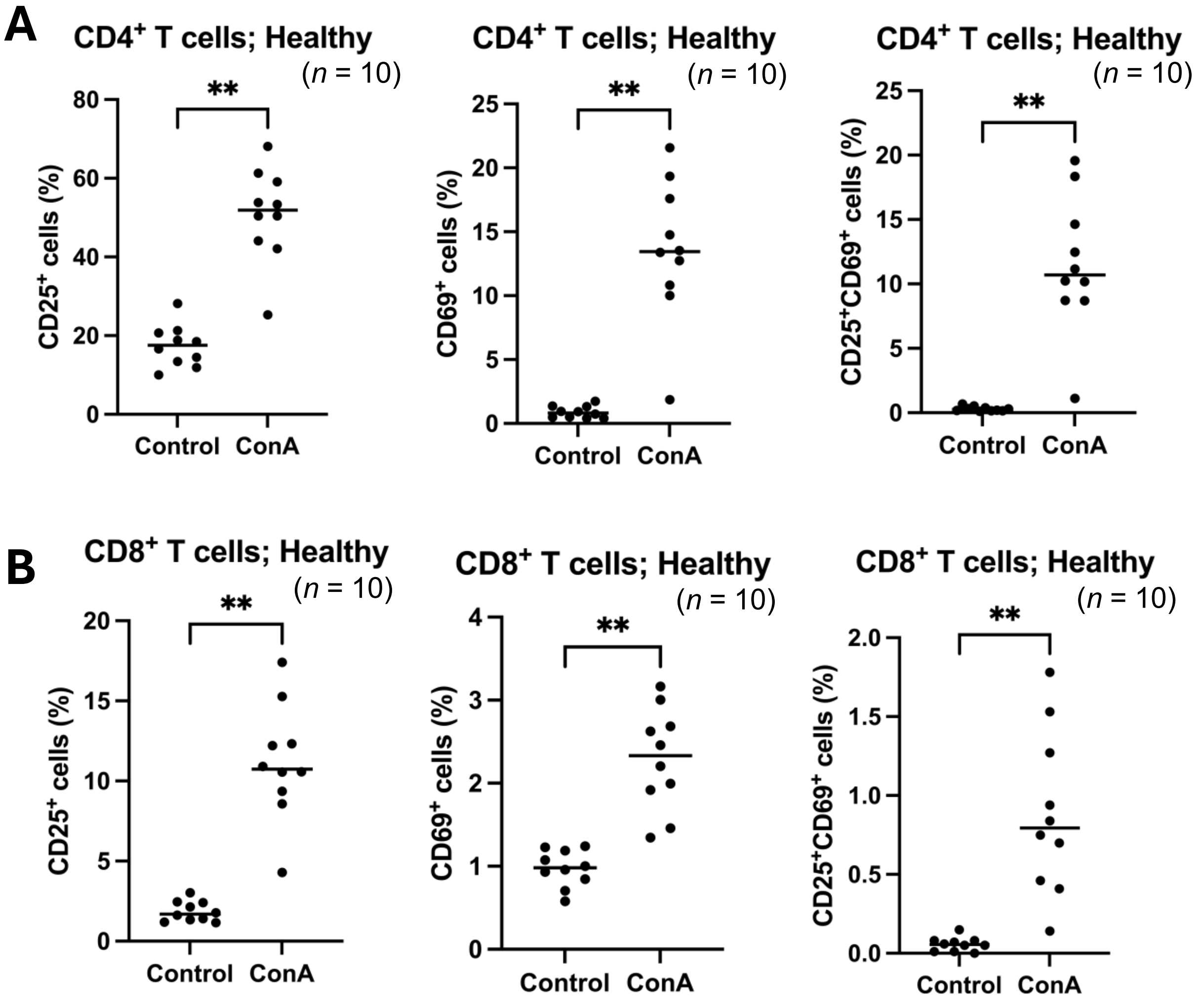

Figure S4. Evaluation of T cell activation marker under ConA stimulation in the cultivation of PBMCs from healthy sheep.

(a, b) Evaluation of $CD25^+$, $CD69^+$, $CD25^+CD69^+$ cell populations in $CD4^+$ (a) and $CD8^+$ subsets (b) in the cultivation assay of PBMCs of BLV-uninfected healthy sheep. Each symbol represents data from an individual animal. Lines indicate median values. Significant differences compared to unstimulated control: **$p < 0.01$.
